# Effects of infant massage on jaundiced neonates undergoing phototherapy

**DOI:** 10.1186/s13052-015-0202-y

**Published:** 2015-11-25

**Authors:** Chien-Heng Lin, Hsiu-Chuan Yang, Chien-Sheng Cheng, Chin-En Yen

**Affiliations:** Divison of Pediatric Pulmonology, China Medical University Children’s Hospital, Taichung, Taiwan; Depatment of Biomedical Imaging and Radiological Science, College of Health Care, China Medical University, Taichung, Taiwan; Department of Early Childhood Development and Education, Chaoyang University of Technology, 168, Jifeng E. Rd., Wufeng District, Taichung, 41349 Taiwan; Department of Pediatrics, Jen-Ai Hospital, Taichung, Taiwan

**Keywords:** Infant massage, Jaundice, Neonates, Phototherapy, Bilirubin

## Abstract

**Background:**

Infant massage is a natural way for caregivers to improve health, sleep patterns, and reduce colic. We aimed to investigate the effects of infant massage on neonates with jaundice who are also receiving phototherapy.

**Methods:**

Full-term neonates with jaundice, admitted for phototherapy at a regional teaching hospital, were randomly allocated to either a control group or a massage group. The medical information for each neonate, including total feeding amount, body weight, defecation frequency, and bilirubin level, was collected and compared between two groups.

**Results:**

A total of 56 patients were enrolled in the study. This included 29 neonates in the control group and 27 in the experimental group. On the third day, the massage group showed significantly higher defecation frequency (*p* = 0.045) and significantly lower bilirubin levels (*p* = 0.03) compared with the control group. No significant differences related to feeding amount or body weight were observed between the two groups.

**Conclusion:**

Infant massage could help to reduce bilirubin levels and increase defecation frequency in neonates receiving phototherapy for jaundice.

## Background

Infant massage, in which babies are massaged soon after birth, is a tradition that is common in India and many other countries. Several studies have reported that infant massage can improve weight gain, sleep patterns, growth and development, and autonomic nervous system functions, and that it can also reduce the rates of colic and infant mortality [1–6]. In addition, massage therapy can help reduce infant stress and can promote positive emotional bonding between parents and babies [7, 8].

Jaundice refers to the yellow staining of the skin and sclerae caused by an increase in serum bilirubin levels [9]. Excessive hyperbilirubinemia can lead to permanent brain damage. Jaundice affects as many as 60 % of healthy neonates and is responsible for 75 % of hospitalizations within the first week after birth [10]. Most cases of neonatal jaundice are caused by unconjugated hyperbilirubinemia, which occurs because of excessive bilirubin formation and because the neonatal liver is unable to clear bilirubin rapidly enough from the blood [10]. This type of jaundice, known as physiological jaundice, is typically harmless; although it should be monitored, it is not likely to require treatment. However, some neonates suffer from exaggerated physiological jaundice or pathological jaundice. These cases should be treated with phototherapy or they may even require exchange transfusions to reduce the risk of acute bilirubin encephalopathy or kernicterus [10, 11].

Previous research has indicated that infant massage decreases the bilirubin level of neonates suffering from hyperbilirubinemia and ameliorates neonatal jaundice [12, 13]. One study reported that full-term infants who received massage therapy had significantly lower serum total bilirubin and transcutaneous bilirubin levels than infants who did not undergo massage therapy [12]. Another study indicated that mean bilirubin levels could be significantly reduced in full-term infants by the fourth day of massage therapy, compared with infants not treated with massage [13]. Despite the fact that earlier clinical studies support the use of massage for reducing neonatal jaundice, the apparent correlation has not been extensively examined among neonates with jaundice who are receiving phototherapy. Therefore, we aimed to evaluate how infant massage affects neonates receiving phototherapy for jaundice.

## Methods

### Study participants

All participants in this study were normal neonates (from birth to 5 days of age) who were receiving phototherapy for jaundice (bilirubin level greater than 15 mg/dL), according to the recommendations of The Society of Neonatology and the standard medical practice in Taiwan. Infants were recruited between August 2011 and July 2012 from a regional teaching hospital in Taichung city, located in central Taiwan.

The inclusion criteria for study participation were as follows: (1) healthy full-term (gestational age, 37–41weeks) neonates, (2) birth weight of 2500–3600 g, (3) APGAR score at birth of 8–10, and (4) receiving phototherapy for hyperbilirubinemia. We excluded infants with rhesus and ABO incompatibility, subgaleal hemorrhage, congenital anomalies, infections, a glucose-6-phosphate dehydrogenase deficiency, gastrointestinal obstruction, and biliary atresia.

The sample size was calculated before the study. The estimated minimum detectable difference of bilirubin in means = 2 mg/dL, expected standard deviation of residuals = 2 mg/dL, 1-β = 0.8, α = 0.05. The minimum sample size of each group was 17 subjects. We divided the participants into either a control group or a massage group by non-blinded, simple randomization. The institutional review board of our hospital approved the study, and informed consent was obtained from the parents of the neonates.

### Phototherapy

In general, healthy full-term infants were discharged at 3 days of age. If neonatal jaundice was found and phototherapy was available in the nursery, they received phototherapy there. However, if phototherapy was not available in the nursery, infants with jaundice were discharged and transferred to another hospital for further care.

Phototherapy was delivered using a halogen lamp (YON DON, YD-P-222; Taiwan) at a distance of 45–60 cm from the neonate, according to the luminosity. The baby’s clothing was otherwise kept to a minimum to expose as much of the neonate’s skin surface to the therapy light as possible. However, the neonate’s eyes and genitalia were covered to prevent damage. When the bilirubin level became less than 10 mg/dL, phototherapy was discontinued.

### Data collection

Demographic information was collected for all children, which included their age, gender, gestational age, and birth weight. We also recorded the following clinical data throughout the study period: (1) total feeding amount, (2) body weight, (3) defecation frequency, and (4) microbilirubin level.

Total feeding amount, body weight, and defecation frequency were recorded from the first to the third day of hospitalization, according to the mothers’ parenting logs and nursing records. Bilirubin was measured using a microassay, which is a rapid, reliable, simple, and accurate estimator of total and direct bilirubin [14]. This test required only minimal blood samples to be taken from the neonate’s foot for analysis in a TBA-80FR analyzer (Toshiba, Tokyo, Japan).

The primary outcome was the termination of phototherapy, and the secondary outcome was discharge after the completion of treatment.

### Massage procedure

All participating neonates in the intervention group received massage therapy from trained therapists. Neonates in the control group did not receive massage therapy. Each massage therapy session started on the first day of phototherapy, lasted for 15–20 min per session, and was conducted twice daily (between meals) for 3 consecutive days. Phototherapy was stopped for the 15–20 min during which neonates received massage therapy.

Massage techniques were performed in accordance with International Association of Infant Massage (IAIM) guidelines. The researcher thoroughly washed his or her hands, applied massage oil (sweet almond oil, AEOMA, England), and then performed a skin test before starting the first massage therapy procedure. For the skin test, we applied sweet almond oil to the inside of baby’s wrist, and after 30 min, we checked the skin for redness, a rash, or other signs of allergic reaction. None of the neonates in the massage group had an allergic reaction or experienced side effects from the sweet almond oil. After the test, we started the massage on the leg and foot (with one hand used to fix the foot), before progressing to the abdomen, hands, and finally, the back. The same researcher provided massage therapy to all neonates in the study, and the room temperature was maintained at between 26 °C and 28 °C.

### Statistical analyses

Data were analyzed using IBM SPSS for Windows, Version 19.0 (IBM Corp., Armonk, NY, USA). Student’s *t-*tests were used to investigate differences between the control and massage group infants with regard to feeding amount, body weight, defecation frequency, and microbilirubin levels. The Kolmogorov–Smirnov test was used to assess the normality of distribution of the investigated parameters (all parameters were shown to be normally distributed). A chi-square test was used to compare the demographic characteristics of infants in the control and massage groups. Results were considered significant at a *p* value of <0.05 and are presented as mean ± standard deviation.

## Results

### Participant characteristics

A total of 60 neonates with jaundice were initially enrolled. However, one neonate was excluded because his bilirubin level exceeded 22 mg/dL on the second day, and he was transferred to a medical center for further care. Another three neonates were excluded because their hospital stays were less 3 days, and their discharge was against medical advice. Therefore, 56 neonates were included in the final study and randomly assigned to the control group (29 neonates; 16 males and 13 females) and the massage group (27 neonates; 11 males and 16 females).

Table 1 shows the medical characteristics of participating neonates. We observed no significant differences between the two groups in terms of type of feeding, gestational age at birth, body weight at birth, body weight on the date of admission, phototherapy duration, or physical weight loss.

**Table 1:** Demographic characteristics of participating neonates

Variable	Item	Massage group (*n* = 27)	Control group (*n* = 29)	*p* value
Sex	Male	11 (40.7) ^a^	16 (55.2)	0.29
	Female	16 (59.3)	13 (44.8)	
Kinds of feeding	Breast feeding	5 (18.5)	5 (17.2)	0.79
	Infant formula	3 (11.1)	4 (13.8)	
	Mixed	19 (70.4)	20 (69.0)	
Hematoma	Yes	4 (14.8)	2 (6.9)	0.35
	No	23 (85.2)	27 (93.1)	
Type of delivery	Vaginal delivery	25 (92.6)	28 (96.6)	0.52
	Cesarean-section	2 (7.4)	1 (3.4)	
Gestational age (weeks)		38.7 ± 0.7 ^b^	39.1 ± 0.9	0.36
Age (hours)		117 ± 56	109 ± 42	0.41
Body height (cm)		50.9 ± 1.7	50.9 ± 1.9	0.93
Body weight at birth (gm)		3069.3 ± 233.3	3174.5 ± 340.6	0.19
Body weight at admission (gm)		2888.9 ± 271.8	3008.2 ± 308.0	0.13
Physiologic weight loss (gm)^c^		180.4 ± 102.3	166.2 ± 114.5	0.63
Percentage of physiologic weight loss (%)		5.9 ± 3.4	5.1 ± 3.4	0.39
Phototherapy duration (hours)		64.8 ± 16.6	69.4 ± 27.3	0.45
D1 microbilirubin level		16.6 ± 1.4	17.1 ± 1.5	0.23
D2 microbilirubin level		14.1 ± 1.2	14.5 ± 1.7	0.38
D3 microbilirubin level		11.0 ± 1.1	11.8 ± 2.0	0.78
Microbilirubin level at the end of phototherapy		9.6 ± 1.6	10.0 ± 1.8	0.19
Rebound microbilirubin level		(*n* = 3) 10.1 ± 2.4	(*n* = 4) 10.6 ± 1.1	0.55
Hospital stay (hours)		78.5 ± 14.0	87.8 ± 24.0	0.08

^a^ Values are n (%)

^b^ Values are means ± standard deviation

^c^ Physiologic weight loss means the body weight loss between at birth and at admission

D1: first day of hospitalization, D2: second day of hospitalization, D3: third day of hospitalization

### Total feeding amount

For all participating neonates, food intake increased through hospitalization. In both groups, food intake was significantly higher on the second and third days of hospitalization than on the first day (*p* < 0.001). However, no significant difference was observed between the groups during hospitalization (Table 2).

**Table 2:** Feeding intake among massage and control group

Parameter	Massage group (*n* = 27)	Control group (*n* = 29)	t value	*p* value
D1 feeding amount (ml)	330.7 ± 111.0 ^a^	330.4 ± 104.4	0.11	0.99
D2 feeding amount (ml)	504.1 ± 79.8	499.0 ± 99.0	0.21	0.83
D3 feeding amount (ml)	558.1 ± 74.3	555.9 ± 85.8	0.1	0.92

^a^ Values are means ± standard deviation

D1: first day of hospitalization, D2: second day of hospitalization, D3: third day of hospitalization

### Body weight

The body weights of all neonates increased through hospitalization, with the body weight being significantly higher on the third than on the first day of hospitalization (*p* = 0.03). However, there was no significant difference between the groups (Table 3).

**Table 3:** Body weight among massage and control group

Parameter	Massage group (*n =* 27)	Control group (*n =* 29)	t value	*p* value
D1 body weight (gm)	2,888.9 ± 271.8^a^	3,008.2 ± 308.0	-1.53	0.13
D2 body weight (gm)	2,968.1 ± 262.7	3,098.3 ± 323.0	-1.65	0.10
D3 body weight (gm)	3,031.9 ± 263.6	3,163.8 ± 342.1	-1.61	0.11

D1: first day of hospitalization, D2: second day of hospitalization, D3: third day of hospitalization

^a^ Values are means ± standard deviation

### Defecation frequency

Table 4 presents the defecation frequencies for the massage and control groups. The defecation frequency significantly increased for all neonates during hospitalization (*p* < 0.001). Although the defecation frequency was not significantly different between the control and massage groups on the first and second days of hospitalization, it was significantly higher in the massage group on the third day (*p* = 0.04).

**Table 4:** Defecation frequency among massage and control group

Parameter	Massage group (n = 27)	Control group (n = 29)	t value	*p* value
D1 defecation frequency (times)	3.1 ± 1.7^a^	3.0 ± 2.0	0.1	0.99
D2 dfecation frequency (times)	5.0 ± 1.5	4.3 ± 1.5	1.72	0.92
D3 defecation frequency (times)	4.6 ± 1.3	3.9 ± 1.3	2.05	0.04^*^

^a^ Values are means ± standard deviation

^*^Values are significantly different between groups using Student’s t test; *P* < 0.05

D1: first day of hospitalization, D2: second day of hospitalization, D3: third day of hospitalization

### Microbilirubin Level

The microbilirubin level was significantly decreased for all participating neonates during hospitalization (*p* < 0.001). Because intravenous infusions can increase the excretion of bilirubin and thereby decrease serum bilirubin levels, we excluded neonates who received intravenous infusions when comparing microbilirubin levels between the massage and control groups. We found no significant differences in the microbilirubin levels between the control and massage groups during the first and second days of hospitalization. However, on the third day of hospitalization, the microbilirubin level was significantly lower in the massage group than in the control group (*p* = 0.03; Table 5).

**Table 5:** Microbilirubine level of neonetes without intravenous hydration between massage and control group

Parameter	Massage group (*n* = 15)	Control group (*n* = 11)	t value	*p* value
D1 microbilirubine level (mg/dL)	15.6 ± 0.9 ^a^	15.9 ± 1.0	-1.35	0.19
D2 microbilirubine level (mg/dL)	13.9 ± 1.2	14.5 ± 0.8	-1.36	0.18
D3 microbilirubine level (mg/dL)	10.8 ± 0.9	12.2 ± 1.8	-2.6	0.03^*^

^a^ Values are means ± standard deviation

^*^Values are significantly different between groups using Student’s t test; *P* < 0.05

D1: first day of hospitalization, D2: second day of hospitalization, D3: third day of hospitalization

## Discussion

### Body weight

Our study indicated that body weight was not significantly different between the massage and control groups. This result is consistent with some reports of previous studies [15–17]. Lee also failed to identify significant differences in weight gain between infants who received massage therapy and control group infants after 4 weeks of treatment [18]. However, the recent literature has indicated that massage therapy can increase weight gain in preterm infants who receive moderate-pressure massage for 10 min three times per day over 5 days [19].

Serrano et al. also demonstrated that massaged infants weighed significantly more than control infants at 2 months of age [20], whereas Yilmaz et al. reported that both body weight and height significantly increased in massaged infants compared with control infants after 2 and 14 weeks of massage therapy [21]. Field et al. showed that preterm neonates who received massage therapy for a 5-day period had greater increases in weight gain, serum insulin levels, and insulin-like growth factor-1 (IGF-1) [16]. In this latter study, the authors speculated that weight gain following massage might have been due to increases in insulin/IGF-1 levels or vagal activity, which, in turn, could have decreased stress and gastric motility, leading to more efficient food absorption [16].

In our study, the lack of a significant increase in body weight gain after massage may have been because of the young age of our participants (average age: 4.9 ± 2.5 days in the massage group; 4.5 ± 1.7 days in the control group). In addition, it is possible that the duration of massage therapy was too short to stimulate the secretion of insulin and IGF-1.

### Defecation frequency

The defecation frequency in the massage group of this study was significantly higher than that in the control group on the third day of massage therapy, which is comparable with the results of previous studies. In the study by Seyyedrasooli et al., the defecation frequency of infants who received massage therapy was significantly higher than that in the control group by the fourth day of therapy [22]. Also, in the study by Chen et al., the mean defecation frequency of the massage group was significantly higher than that of the control group on the first 2 days of therapy [12]. However, neither of these study population included neonates receiving phototherapy for jaundice.

Previous research has indicated that most neonates first pass feces within 24 h of birth, although massage therapy can stimulate the passage of meconium [12]. This may explain the significantly higher defecation frequency that we observed in the massage group by the third day of treatment. Massage therapy can increase bowel movements and the excretion of meconium [22], and an increased frequency of bowel movements might be expected to diminish the enterohepatic circulation of bilirubin in a neonate, thereby leading to increased bilirubin excretion [23].

### Microbilirubin level

One previous study has suggested that early intravenous nutrition could improve neonatal jaundice by increasing urinary excretion and the metabolism of bilirubin [24]. Therefore, we excluded those neonates receiving intravenous hydration when comparing the bilirubin levels between the massage and control groups. In our study, the bilirubin level of the massage group neonates was significantly lower than that of the control group on the third day of massage therapy. This result is consistent with the study by Chen et al., who reported that in full-term neonates with jaundice, bilirubin levels were significantly decreased in the massage group compared with the control group on the fourth day of therapy [12]. In addition, Moghadam et al. indicated that the mean bilirubin level of infants with jaundice in the massage group significantly decreased on the fourth day compared with the control group [13].

In contrast to these reports, one study has indicated that transcutaneous bilirubin levels in healthy, full-term infants were not significantly different between the massage and control groups after 4 days of therapy [22]. Possible explanations for this inconsistency include differences in the massage procedure and the fact that their neonates had no hyperbilirubinemia. Massage therapy could lead to earlier reductions in bilirubin levels that might allow shorter treatments with phototherapy, resulting in earlier discharge.

The most likely mechanism underlying the reduction in neonatal jaundice in the group receiving massage therapy is the stimulation of intestinal movement. This, in turn, will increase defecation frequency and allow the neonate to pass greater amounts of meconium, which contains bilirubin [12]. This is consistent with the findings of Gourley et al., who noted that stool production and serum bilirubin levels were negatively correlated in healthy term infants during the first week of life [25]. In our study, the defecation frequency was significantly higher in the massage group than in the control group on the third day of treatment. Increased defecation might therefore explain the significant reduction in bilirubin levels observed in the massage group.

Moreover, massage therapy also stimulates the vagus nerve, which will increase the frequency of bowel movements and diminish the enterohepatic circulation of bilirubin, thereby increasing bilirubin excretion [22]. Furthermore, in subcutaneous tissue, physiological massage therapy can increase the flow of blood, lymph, and tissue fluids, which increases the collection and excretion of waste products such as bilirubin [12].

Nonetheless, in the current study, the number of participating neonates was small and the duration of massage therapy was short, and these limitations may have affected the reliability of statistical tests or masked other important correlations. Future research should investigate the use of massage therapy over longer periods. We also recommend that neonates receive continuous follow-up, such as home visits by a qualified healthcare professional, to ensure that massage therapy achieves the desired effects.

## Conclusion

This study shows that by the third day of intervention, the defecation frequency of neonates receiving phototherapy for jaundice was significantly higher in those also receiving massage therapy, compared with the control group not receiving massage therapy. Furthermore, microbilirubin levels were significantly lower in the massage group on the third day, but this was only assessed in those who did not receive intravenous hydration. Massage therapy is a safe and economic and no significant harmful practice that can promote bonding and interaction between the mother and infant. However, the benefits of massage therapy remain uncertain for neonatal jaundice, and although this study adds to the evidence in its favor, further research is needed to clarify the true effects of such auxiliary treatments on the outcomes of neonatal jaundice.
